# Old drug repositioning and new drug discovery through similarity learning from drug-target joint feature spaces

**DOI:** 10.1186/s12859-019-3238-y

**Published:** 2019-12-27

**Authors:** Yi Zheng, Hui Peng, Xiaocai Zhang, Zhixun Zhao, Xiaoying Gao, Jinyan Li

**Affiliations:** 1 Faculty of Engineering and Information Technology, University of Technology Sydney, 15 Broadway Ultimo, Sydney, 2007 Australia; 2 School of Engineering and Computer Science, Victoria University of Wellington, Cotton Building, Kelburn Campus, Wellington, 6140 New Zealand

**Keywords:** Drug target prediction, Reliable negative samples, Pairwise similarity

## Abstract

**Background:**

Detection of new drug-target interactions by computational algorithms is of crucial value to both old drug repositioning and new drug discovery. Existing machine-learning methods rely only on experimentally validated drug-target interactions (i.e., positive samples) for the predictions. Their performance is severely impeded by the lack of reliable negative samples.

**Results:**

We propose a method to construct highly-reliable negative samples for drug target prediction by a pairwise drug-target similarity measurement and OCSVM with a high-recall constraint. On one hand, we measure the pairwise similarity between every two drug-target interactions by combining the chemical similarity between their drugs and the Gene Ontology-based similarity between their targets. Then we calculate the accumulative similarity with all known drug-target interactions for each unobserved drug-target interaction. On the other hand, we obtain the signed distance from OCSVM learned from the known interactions with high recall (≥0.95) for each unobserved drug-target interaction. After normalizing all accumulative similarities and signed distances to the range [0,1], we compute the score for each unobserved drug-target interaction via averaging its accumulative similarity and signed distance. Unobserved interactions with lower scores are preferentially served as reliable negative samples for the classification algorithms. The performance of the proposed method is evaluated on the interaction data between 1094 drugs and 1556 target proteins. Extensive comparison experiments using four classical classifiers and one domain predictive method demonstrate the superior performance of the proposed method. A better decision boundary has been learned from the constructed reliable negative samples.

**Conclusions:**

Proper construction of highly-reliable negative samples can help the classification models learn a clear decision boundary which contributes to the performance improvement.

## Background

Detection of drug-target interactions plays a vital role in both old drug repositioning and new drug discovery. It helps to identify new targets for existing drugs or predict new drugs for known targets. Currently, only a small number of drug-target interactions are validated via wet-lab experiments. A large proportion of interactions remain to be investigated by computational algorithms due to the high monetary and time cost of wet-lab experiments.

Some specially designed machine-learning methods have been proposed recently in this research domain to overcome the challenging issues. These methods can be classified into three major categories: similarity-based methods, feature vector-based methods and other methods. The similarity-based methods are all guided by the “guilt-by-association” assumption that similar targets tend to be targeted by similar drugs and vice versa [1]. Ding et al. [2] had a comprehensive review on similarity-based machine learning methods. Models including nearest neighbor [3], kernelized Bayesian matrix factorization [4], network-based inference [5], bipartite local models [3], gaussian interaction profile [6], and pairwise kernel method (PKM) [7] are summarized briefly and computationally compared in their work. The comparison results show that PKM performed the best in terms of AUC (area under the receiver operating characteristic curve).

In the feature vector-based methods, each drug-target pair (DTP) is represented as a fixed-length feature vector. The feature vector is encoded by various types of properties of drugs and targets, such as drug chemical structures and target sequences. For example, using the method proposed by Yu et al. [8], each drug is represented as a 1080-feature vector consisting of constitutional descriptors, topological descriptors, 2D correlations, molecular properties and etc. Likewise, each protein is transformed into a 1080-dimension feature vector. Merging them together, a set of 2160 features is taken to describe the drug-protein pairs for the Random Forest predictor. Luo et al. [9] developed DTINet, a computational pipeline which integrates diverse drug-related information from heterogeneous data sources. DTINet can learn well from low dimensional vector representations for accurate interpretation of the topological properties of nodes in the heterogeneous network. Then, DTINet makes predictions based on these representations via a vector space projection scheme.

Apart from detecting the drug-target interactions using similarity information or feature vector-based representation, researchers also attempted to use other information such as bio-medical documents for detection. Zhu et al. [10] proposed a probabilistic model named MAM to mine drug-gene relations from literature. MAM is composed of a mixture of aspect models, each of which is designed for one type of co-occurrence data and its learning algorithm. Their experimental results show that the prediction performance is improved via combining different types of co-occurrence data. Although potential drug-target interactions can be mined from the bio-medical documents, they have significant drawbacks such as low data quality and incompetency for novel relations.

These existing machine-learning approaches use the experimentally validated DTPs as positive samples, and use all or a random subset of unobserved DTPs as negative samples for the training of the classification models [3, 4, 6, 7]. As suggested by Ding [2], such negative samples might include potential drug-target interactions not yet known, and would unavoidably result in inaccurate predictive results. Because the current machine-learning methods are severely impended by the lack of reliable negative samples, we develop a method to identify highly reliable negative samples of DTPs to improve the prediction performance.

Based on the “guilt-by-association” assumption that similar drugs tend to interact with similar targets, the existing methods have achieved remarkable performance. Thus it is also reasonable to select reliable negative samples based on its converse negative proposition, i.e., a drug dissimilar to all drugs known to interact with a target is less likely to bind the target and vice versa.

One-class Support Vector Machine (OCSVM) [11] has demonstrated its advantages for classification in the absence of positive or negative samples [12]. It learns a hypersphere from the training data, ensuring most training data are in the hypersphere. OCSVM requires one-class data only, thus it is an ideal technique to identify reliable negatives (i.e., outliners) for drug-target prediction where only positives are available.

In this work, we propose a method to construct highly-reliable negative samples for drug target prediction by a pairwise drug-target similarity measurement and OCSVM with a high-recall constraint. On one hand, we measure the pair-wise similarity between every two drug-target interactions by combining the chemical similarity between their drugs and the Gene Ontology-based similarity between their targets. Then we calculate the accumulative similarity with all known drug-target interactions for every unobserved drug-target interaction. On the other hand, we obtain the signed distance using OCSVM learned from the known interactions with high recall (≥0.95) for each unobserved drug-target interaction. Unobserved DTPs with lower accumulative similarities or lower signed distances are less likely to be positives, thus of high-probability to be negatives. Consequently, we compute the score for each unobserved drug-target interaction via averaging its accumulative similarity and signed distance after normalizing all accumulative similarities and signed distances to the range [0,1]. Unobserved interactions with lower scores are preferentially served as reliable negative samples for the classification algorithms. The specific negative number is determined by the negative sample ratio which will be discussed in the experiment section.

In the performance evaluation, we investigated impact of the ratio levels of negative samples on the prediction. We also demonstrated that the performance improvement brought by the reliable negative samples can be achieved for four different classical classifiers and for a domain specially designed prediction model (the pairwise kernel method PKM). Extensive experiments further show that the performances of all models have been improved significantly owing to the use of reliable negative samples.

## Methods

### Prediction framework

**Fig. 1:**
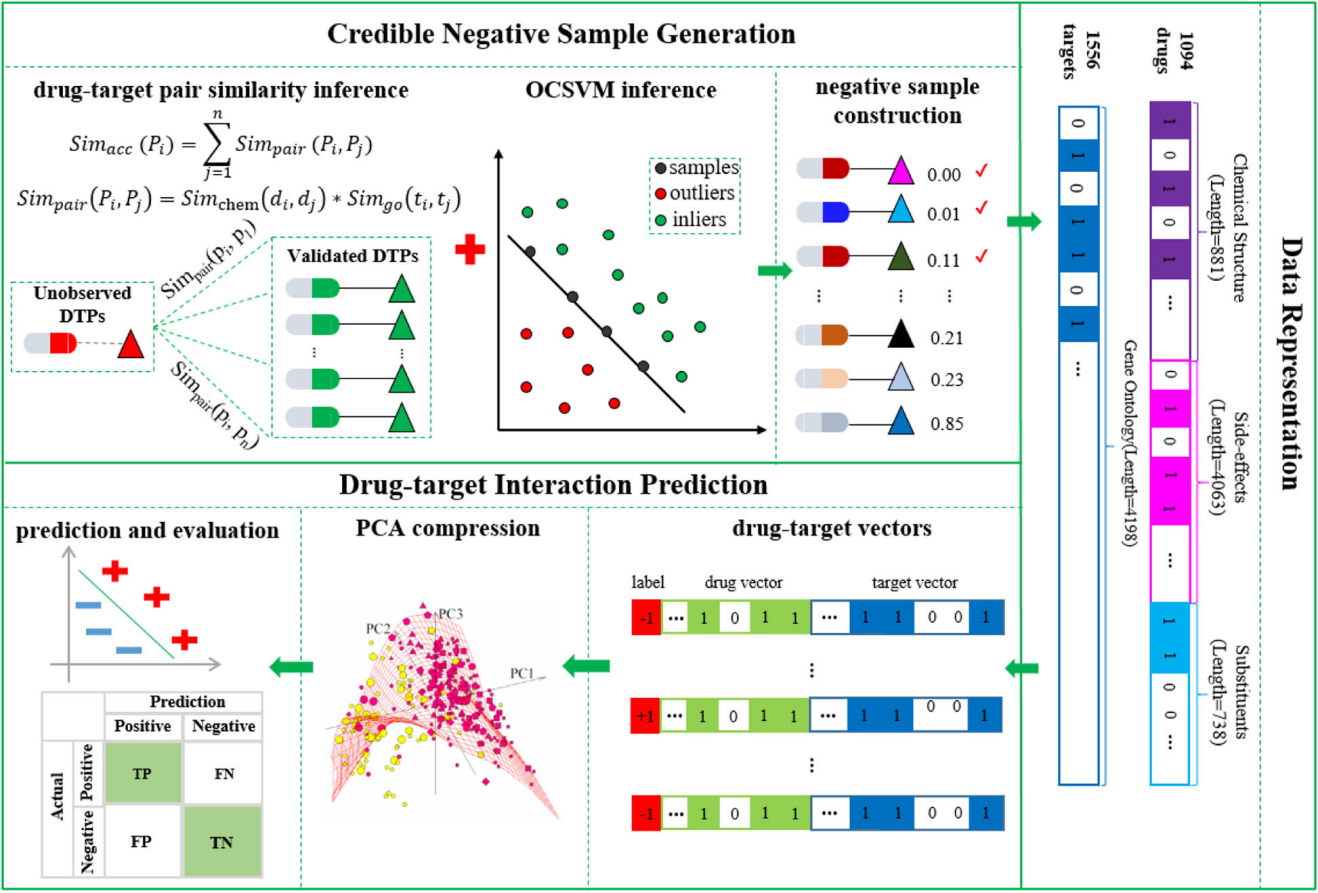
Framework of the proposed method. It consists of three components: credible negative sample generation, data representation, and drug-target interaction prediction. DTPs: drug target pairs; OCSVM: one-class support vector machine; PCA: principle component analysis

The prediction framework is illustrated in Fig. 1. It consists of three main components: credible negative sample generation, data representation, and drug-target interaction prediction. First, unobserved DTPs are ranked in ascending order of their scores computed by the pair-wise similarity and OCSVM. A corresponding number of them are sequentially selected to construct a reliable negative sample set. Then drugs and targets are represented as 5682-dimensional and 4198-dimensional vectors respectively according to their properties. Drug-target vectors can be obtained by appending the target vector to the drug vector together. Following that, PCA (principal component analysis) is performed to reduce the dimension of raw drug-target vectors. Finally, truncate drug-target vectors with their labels are used to train the classifier for subsequent predictions.

### Credible negative sample generation

**Fig. 2:**
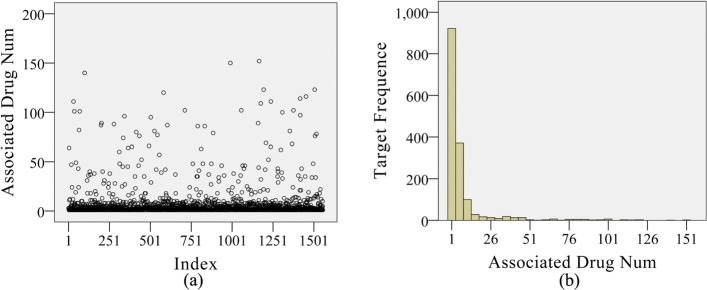
Characteristics of targets and their associated drugs. The left panel (**a**) is the index-plot of the number of associated drugs for each target and the right panel (**b**) is the histogram of the associated drug number for the targets

It can be observed from Fig. 2 that a great number of targets only interact with one drug. It is indicative that there are abundant unobserved DTPs. Among these unobserved DTPs, some should be true interactions (positive samples) which are yet unobserved. Therefore, treating these unobserved DTPs all as negative samples by the traditional methods is unreasonable which may cause more false classifications [13]. A method to construct a reliable negative sample set becomes vital to achieve precise predictions.

Most existing machine-learning approaches developed for drug-target interaction prediction are based on the assumption that similar drugs tend to bind similar targets and vice versa. Consequently, it is reasonable to select reliable negative samples based on its converse negative proposition that drugs dissimilar to all drugs known to bind a target are less likely to interact with the target and vice versa.

In this work, we propose to combine the converse negative proposition of the guilt-by-association methods and the power of OCSVM to construct reliable negative samples. On one hand, we infer the probabilities of unobserved DTPs to be negatives by a pairwise drug-target similarity measurement. To be specific, we first measure the similarities between drugs according to their chemical structures. Each drug is represented as a 1024-dimensional fingerprint using the open-source tool CDK (Chemistry Development Kit) [14]. Formally for a drug *d*, it is represented as $f^{d}\left (f_{i}^{d}\in \{0,1\}, i\in \{1,2,...,1024\}\right)$. Then the chemical similarity between two drugs, say drug *d*_*i*_ and drug *d*_*j*_, is calculated by their Tanimoto score:

1 $$ {Sim}_{chem}(d_{i}, d_{j})=\frac{\sum_{l=1}^{1024}\left(f_{l}^{i}\land f_{l}^{j}\right)}{\sum_{l=1}^{1024}\left(f_{l}^{i}\lor f_{l}^{j}\right)},  $$

where ∧ and ∨ are bit-wise “and” and “or” operators respectively; $f_{l}^{i}$ and $f_{l}^{j}$ are the *l*^*th*^ bit of fingerprints of drug *d*_*i*_ and drug *d*_*j*_ respectively. We also measure the similarity between two target proteins as the overlapping ratio of their related GO terms. Suppose *GO*^*i*^ and *GO*^*j*^ are the GO term sets for the target protein *t*_*i*_ and *t*_*j*_ respectively, the similarity score between *t*_*i*_ and *t*_*j*_ is defined as:

2 $$ {Sim}_{go}(t_{i}, t_{j})=\frac{GO^{i} \cap GO^{j}}{GO^{i} \cup GO^{j}},  $$

where ∩ and ∪ are “intersection” and “union” operators respectively. Then, we measure the pairwise similarity between two DTPs by combining the drug similarity and the target protein similarity. The pairwise similarity between the drug-target pair *p*_*i*_(*d*_*i*_−*t*_*i*_) and *p*_*j*_(*d*_*j*_−*t*_*j*_) is given by:

3 $$ {Sim}_{pair}(p_{i}, p_{j}) = {Sim}_{chem}(d_{i}, d_{j}) * {Sim}_{go}(t_{i}, t_{j}).  $$

Following that, we calculate the accumulative pairwise similarity with all the validated DTPs for each unobserved DTP. For an unobserved DTP *p*_*i*_, its accumulative pairwise similarity is measured by:

4 $$ {Sim}_{acc}(p_{i}) = \sum_{j=1}^{n} {Sim}_{pair}(p_{i}, p_{j}),  $$

where *n* is the total number of validated DTPs.

On the other hand, we infer the probabilities by OCSVM. Specifically, we use signed distances which denote the distances between the unobserved DTPs and the calculated OCSVM separating hyperplane to measure their probabilities (obtained using sklearn.svm.OneClassSVM.decision_function of the Python scikit-learn package). We feed OCSVM with all known DTPs and optimize its parameters via 5-fold cross-validation. A high recall constraint (≥0.95) is required to ensure that the majority of true DTPs are correctly predicted. With the optimized parameter settings (*nu*: 0.1, *gamma*: 0.05, recall=0.96), we obtained the signed distances for all unobserved DTPs.

After we get the accumulative pairwise similarities and signed distances for all DTPs, we normalize them to the range [0,1] via the formula 5 and 6 respectively.

5 $$ {NSim}_{acc}(p_{i}) = \frac{{Sim}_{acc}(p_{i}) - {Sim}_{acc}^{min}}{{Sim}_{acc}^{max} - {Sim}_{acc}^{min}},   $$

where ${Sim}_{acc}^{max}$ and ${Sim}_{acc}^{min}$ are the maximum and minimum value of all accumulative pairwise similarities respectively, *NSim*_*acc*_(*p*_*i*_) and *Sim*_*acc*_(*p*_*i*_) are the normalized and raw accumulative pairwise similarity for DTP *p*_*i*_.

6 $$ {NDis}_(p_{i}) = \frac{Dis(p_{i}) - {Dis}_{min}}{{Dis}_{max} - {Dis}_{min}},   $$

where *Dis*_*max*_ and *Dis*_*min*_ are the maximum and minimum value of all signed distances, *NDis*(*p*_*i*_) and *Dis*(*p*_*i*_) are the normalized and raw signed distance for DTP *p*_*i*_.

The “guilt-by-association” methods assume that similar drugs are more likely to interact with similar targets [2]. Consequently, unobserved DTPs with lower accumulative similarities are less likely to be true positives and of high-probability to be true negatives. OCSVM predicts DTPs with higher normalized signed distances as positives, thus unobserved DTPs with lower normalized signed distances are more likely to be true negatives. Consequently, it’s reasonable to combine the above two factors as a single probability score as follows: *Score*(*p*_*i*_)=(*NSim*_*acc*_(*p*_*i*_)+*NDis*(*p*_*i*_))/2. Finally, we rank all unobserved DTPs in ascending order of their probability scores (**screen negative list**, see Additional file 1), and those with lowest scores are taken to form the set of negative samples. The specific number is determined by the negative sample ratio which is discussed in the experiment section.

### Data representation via vectors

To perform the machine-learning task, we represent drugs and target proteins as vectors according to their properties. Specifically, each drug is represented as a 5682-dimensional binary vector using its chemical substructures (881), side-effects (4063) and substituents (738). The elements of the drug vector encode for the presence or absence of each property (i.e., chemical substructures/side-effects/substituents) by 1 or 0. The drug chemical substructures correspond to the 881 chemical substructures defined in PubChem [15]. The side-effects and substituents are 4063 unique side-effects from SIDER [16] and 738 unique substituents from Drugbank [17, 18] respectively. Likewise, each protein is represented as a 4198-dimensional binary vector where each bit denotes the presence or absence of the unique GO term by 1 or 0. Finally, we obtain the vector of any drug-target pair by appending the target vector to the drug vector.

### Prediction of drug-target interactions

The dimension of each DTP vector is 9880 (5682 + 4981) and there are 1,702,264 (1,094*1,556) possible DTPs between 1094 drugs and 1556 targets used for experiments. Thus the size of the classification input could be around the order of magnitude of billion (9,880*1,702,264). Such high dimensionality will inevitably incur a huge time and computational cost. In this study, we employ PCA to map raw vectors of DTPs into lower-dimension space to speed up the prediction process. To be specific, we fit PCA with all training DTP vectors first. Then we transform both the training and test DTP vectors into lower-dimensional vectors. The PCN (principle component number) is set as 225 and the specific determining process is described in Additional file 2: Figure S2.

We label all positive samples (i.e., experimentally validated DTPs) as +1 and the reliable negative samples as -1. The compressed vectors of DTPs together with their labels are used to train a binary classifier (e.g., Random Forest) for subsequent prediction. The prediction performance is evaluated via 5-fold cross validation: (1) samples in the gold standard are split into 5 roughly equal-sized subsets; (2) each subset is taken in turn as the test set, and the remaining subsets are used as training set; (3) all results over the 5-fold validation are used for evaluation. Evaluation metrics widely used in binary classification including AUC, precision, recall, and F1-Score are employed to demonstrate the prediction performance.

## Results and discussions

In this section, we first describe the details of the data used in this work. Then we investigate impacts of the ratio levels of negative samples to the positive samples on the prediction performance. Using the best setting for the negative sample ratio, we then evaluate the performance improvement brought by the reliable negative samples by four classical classifiers. Finally, we further demonstrate the superior performance of the proposed method using PKM, a state-of-the-art predictive method proved to be the most powerful in Ding’s review [2].

### Data resources

We use the benchmark dataset collected by Zheng et al. [19] for experiments. It consists of 1094 drugs and 1556 targets. Drug properties including chemical structures and substituent are extracted from DrugBank [17, 18], a comprehensive drug database. All side-effects are downloaded from SIDER [16] and the GO terms of target proteins are retrieved from the EMBL-EBI website [20]. The statistical details of the data sources are summarized in Table 1. The distribution of the experimentally validated drug-target interaction pairs is illustrated in Fig. 2. Information of all researched drugs, targets and validated DTPs is available in Additional file 3. All the above data and the source codes are included in Additional file 4.

**Table 1:** Statistical details of the dataset used in this work

Field	Value
Number of drugs	1094
Number of targets	1556
Number of validated interacted DTPs	11,819
Number of unique side-effects	4063
Number of unique substituents	738
Number of unique GO terms	4198

### Impacts of negative sample ratio levels on the prediction performance

**Fig. 3:**
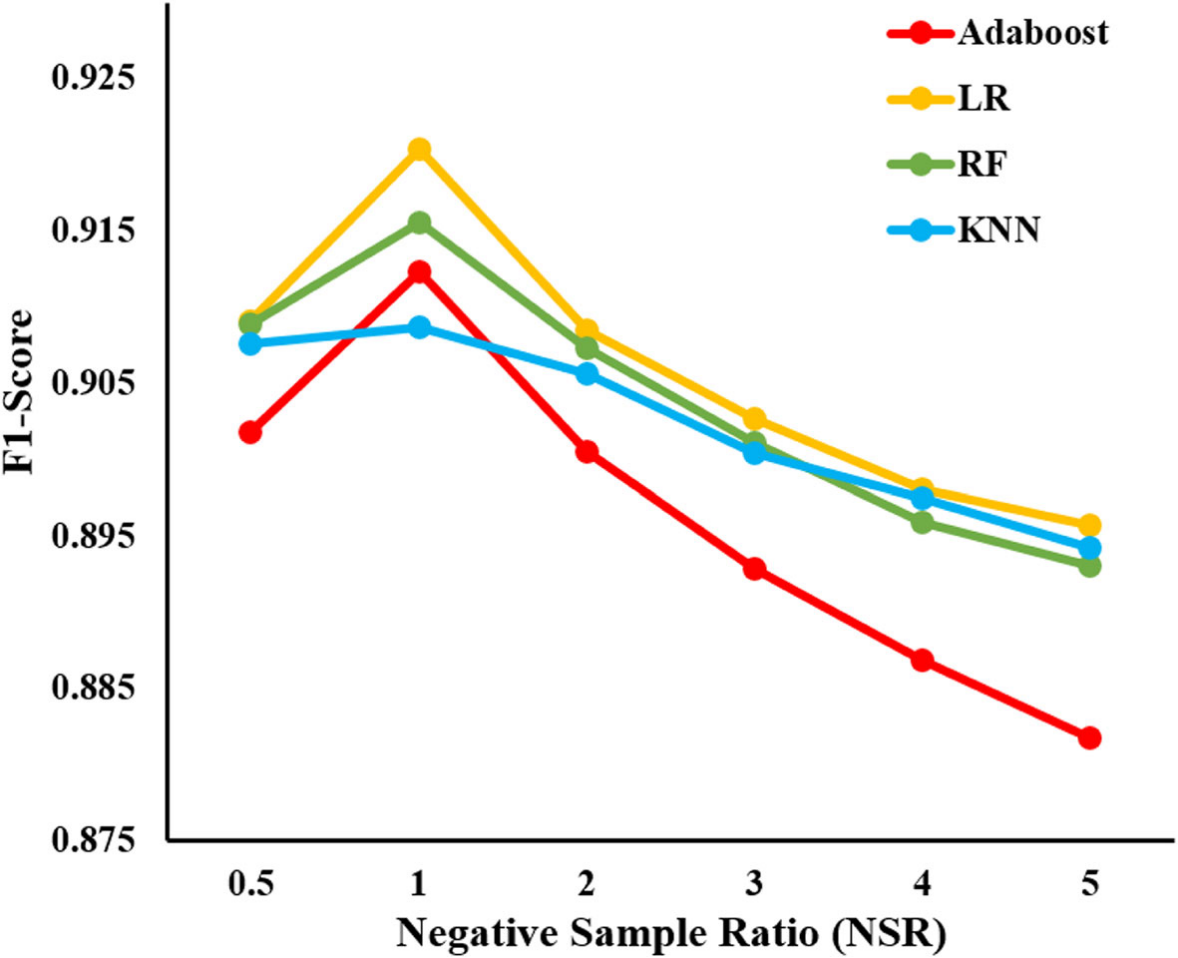
The F1-Scores of four classifiers on reliable negative samples with different negative sample ratio levels. The x-axis is the negative sample ratio and the y-axis is the F1-Score

There are 11,819 experimentally validated interactions between the 1094 drugs and the 1556 target proteins used in this work. The remaining 1,690,445 (1094*1556 - 11,819) DTPs are unobserved DTPs, about 143 times the number of validated DTPs. It is impossible to take all unobserved DTPs as negative samples for prediction. In this work, we take all validated DTPs as positive samples. Similar to [21], we investigate how the performance varies when the ratio of negative samples (ratio relative to positive samples) increases from 0.5 to 5. The negative samples are sequentially extracted from the screen negative list (see “Credible negative sample generation” section). Four classical classifiers including Adaboost, LR (logistic regression), KNN (k-nearest neighbor) and RF (random forest) are employed for the training and prediction. All the classifiers are implemented using Python 2.7.13 (sklearn) with the default settings. The F1-Scores achieved by these classifiers under different levels of negative sample ratios are depicted in Fig. 3. It can be seen that the prediction performance of all the four classifiers increases a bit with the negative sample ratio 0.5. Then the performance begins to decrease when the negative sample ratio is larger than 1. The same trend can be observed from the AUC shown in Additional file 2: Figure S1. The training time increases with the increasing number of training samples. Considering the prediction performance and time cost, we take 1 as the optimized negative sample ratio in the following experiments.

### Much better performance than using accumulative pairwise similarity alone and randomly generated negative samples

**Fig. 4:**
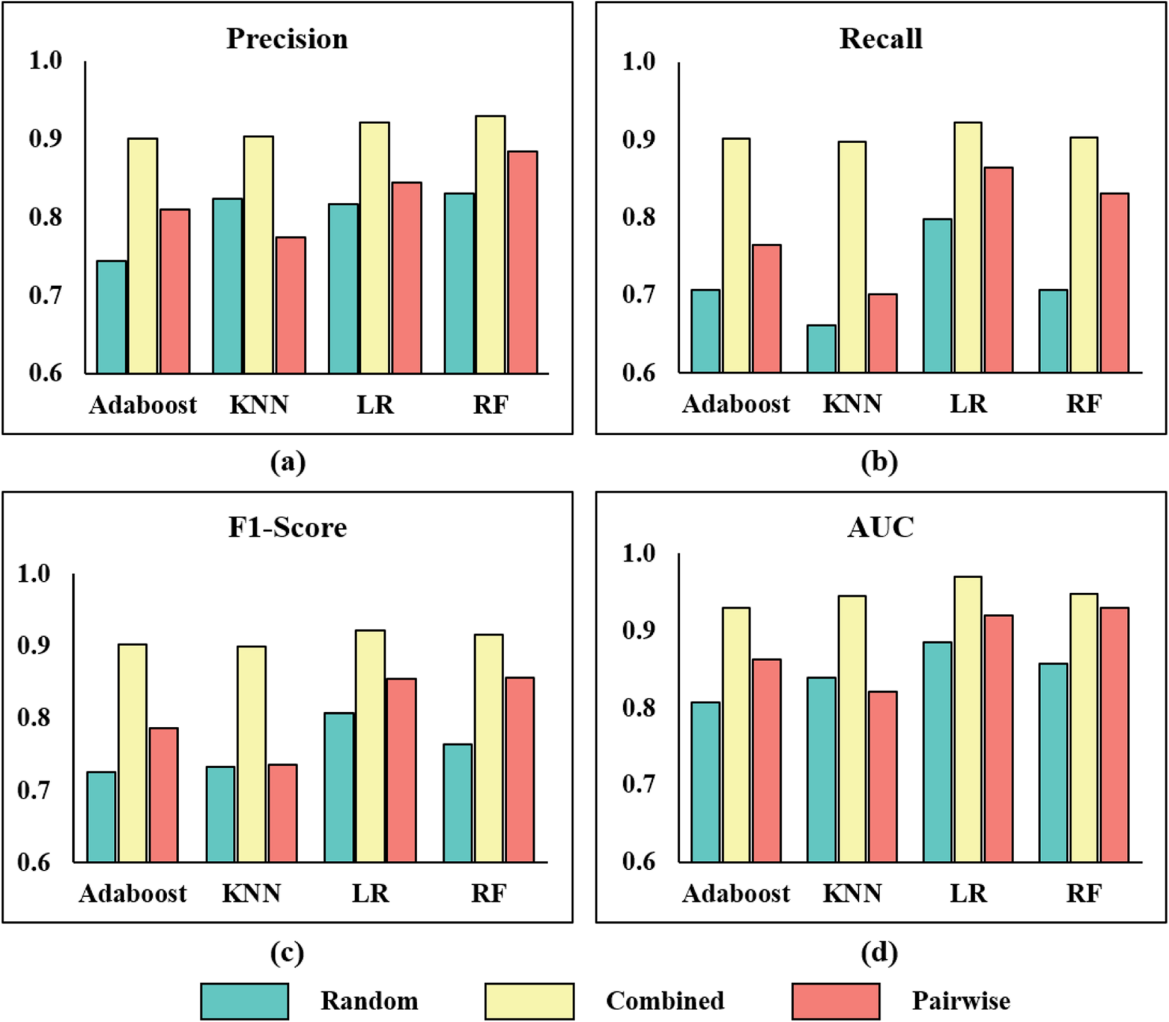
Histograms of precision/recall/F1-Score/AUC values for four classical classifiers on reliable, pairwise, and randomly generated negative samples. Panel (**a**) shows the precision, panel (**b**) shows the recall, panel (**c**) denotes the F1-Score and panel (**d**) is the AUC

To demonstrate the advantage of incorporating signed distances to accumulative pairwise similarities and the prediction performance improvement brought by the constructed reliable negative samples (**Reliable**, negatives sequentially extracted from the screen negative list), we compare them with negative samples inferred by accumulative pairwise similarities alone (**Pairwise**) and randomly generated negative samples (**Random**). The negative samples inferred by the accumulative pairwise similarities are negatives sequentially extracted from DTPs in ascending order of their accumulative pairwise similarities. The randomly generated negative samples are obtained by randomly sampling DTPs which are not in the positive samples. Apart from the negative samples, other settings are the same (NSR = 1). To avoid bias, **Random** is repeated 5 times and the average results are used for the final evaluation. The bar chart of the results are presented in Fig. 4 and the specific values are listed in Additional file 3: Table S1. It can be observed from Fig. 4 that all the four classifiers achieve significantly better performance on all the evaluation indices when using the reliable negative samples (colored yellow) than using negative samples inferred by the accumulative pairwise similarities (colored orange) and randomly generated negative samples (colored green). For example, Adaboost, KNN, Logistic Regression, and Random Forest’s F1-Score improvements are 24.38%, 22.75%, 14.14% and 19.92% over **Random** respectively, and 14.6%, 22.35%, 7.82% and 6.89% over **Pairwise** respectively. Besides, with **Pairwise**, Adaboost, KNN, LR and RF achieves 8.5%, 0.3%, 5.86% and 12.19% F1-Score improvements over **Random** respectively. The above results show that the proposed pairwise similarity and its combination with the OCSVM signed distances contribute the performance improvement. Better classification boundary has been successfully learned from the constructed reliable negative samples by these classifiers.

### Significant improvement for the domain predictive method

**Fig. 5:**
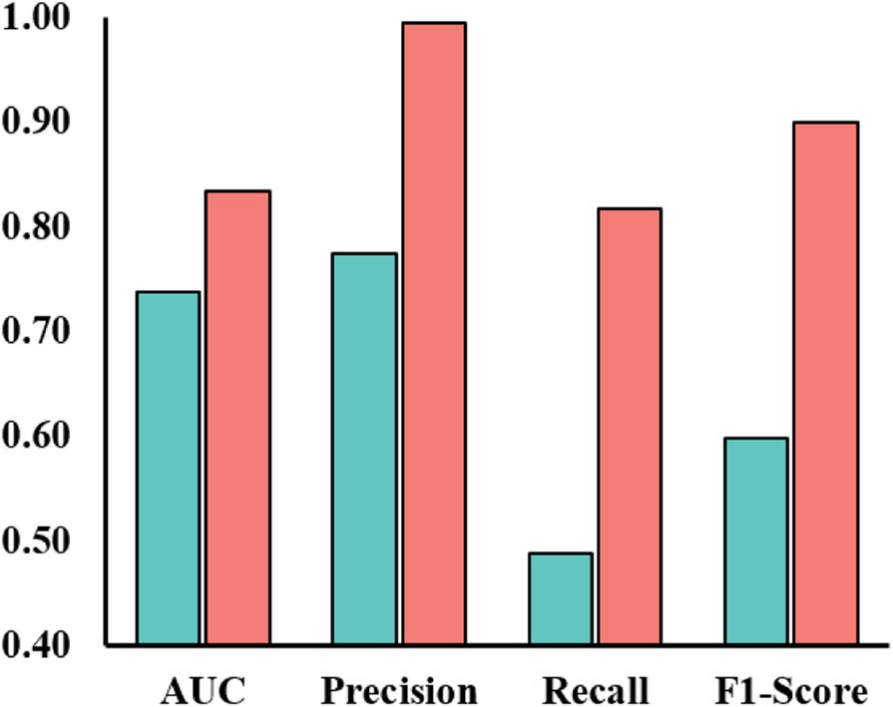
AUC/Precision/recall/F1-Score values of PKM on reliable and randomly generated negative samples

To further confirm the superior prediction performance when using the reliable negative samples, we investigated whether the existing domain predictive methods can achieve better performance. Specifically, we conducted experiments for the domain prediction method PKM (pairwise kernel method), which was suggested to be the most powerful prediction method in Ding’s review [2]. PKM first computes the pairwise similarity between two drug-target pairs as follows:

7 $$ {sim}_{p}\left((d,t),(d^{\prime},t^{\prime})\right)= {sim}_{d}(d,d^{\prime})*{sim}_{t}(t,t^{\prime}),  $$

where *sim*_*d*_ and *sim*_*t*_ are the drug similarity and target similarity (drug chemical structure similarity and target GO similarity used in this work) respectively. Then PKM trains an SVM (support vector machine) with the pairwise similarity kernel to predict scores of arbitrary drug-target pairs. As mentioned in the “Impacts of negative sample ratio levels on the prediction performance” section, we set the negative sample ratio as 1. We compare the prediction performance of PKM when it used the reliable negative samples or when it used randomly selected negative samples (the default setting of PKM). The results are shown in Fig. 5. We can see that the performance of PKM is improved on all the indices when using the reliable negative samples. In detail, the improvements on precision, recall, F1-Score and AUC are significant at 22.1%, 40.3%, 33.4% and, 11.4% respectively. The result reveals that training with the reliable negative samples, PKM learned a better decision boundary indeed for a significant overall improvement on prediction performance.

## Conclusions

In this work, we propose to improve drug-target predictions by constructing highly reliable negative samples by a pairwise drug-target similarity measurement and OCSVM (one-class support vector machine) with a high-recall constraint. On one hand, we measure the pair-wise similarity between every two drug-target interactions by combining the chemical similarity between their drugs and the Gene Ontology-based similarity between their targets. Then we calculate the accumulative similarity with all known drug-target interactions for each unobserved drug-target interaction. On the other hand, we obtain the signed distance using OCSVM learned from the known interactions with high recall (≥0.95) for each unobserved drug-target interaction. After normalizing all accumulative similarities and signed distances to the range [0,1], we compute the score for each unobserved drug-target interaction via averaging its accumulative similarity and signed distance. Unobserved interactions with lower scores are preferentially served as reliable negative samples for the classification algorithms. In the experiment, we investigated how the negative sample ratio level impacts on the prediction performance first. Then we evaluated the performance improvement brought by the constructed negative samples comparing with the case of training on the random negative samples. The comparison experiments were conducted for four classical classifiers and a domain specifically designed predictive model PKM. The extensive experiments demonstrate that the prediction performance has been improved significantly owing to the constructed highly-reliable negative samples.

The proposed method is valuable to both old drug re-positioning and new drug discovery. It can guide and speed up the laborious, expensive and tedious experimental identification of drug-target interactions [22]. In this work, drug chemical structures and protein related GO terms are employed to measure the similarity between drugs and target proteins respectively. We note that more information about drugs (e.g., side-effects, substituents) and target proteins (e.g., protein sequences) can be utilized to measure more of their similarities. This is an interesting problem which will be studied in our future work.

## Supplementary information

**Additional file 1** Unobserved drug-target pairs with their inference scores (ranked in the ascending order).

**Additional file 2** The supplementary figures for this work.
∙ Figure S1: The AUC scores of four classifiers on reliable negative samples with different negative sample ratio levels.
∙ Figure S2: F1-scores of the proposed method with different PCNs (principle component numbers). The x-axis is the PCA component number and the y-axis is the F1-score.

**Additional file 3**
∙ Table S1: AUC/Precision/recall/F1-Score values of four classical classifiers when using reliable, pairwise or randomly generated negative samples.
∙ Table S2: 1094 drugs researched in this work.
∙ Table S3: 1556 targets researched in this work.
∙ Table S4: 11,819 validated drug-target interactions.

**Additional file 4** Supplementary codes and data. The Python codes of the proposed method and the source data.

## Data Availability

The data used in this study all are available in the Additional files.

## Abbreviations

AUC: Area under the receiver operating characteristic curve
CDK: Chemistry development kit
DTP: Drug target pair
KNN: K-nearest neighbor
LR: Logistic regression), OCSVM: One-class support vector machine
PCN: Principle component number
PKM: Pairwise kernel method
RF: Random forest
SVM: Support vector machine
