# Supplementary material for: Old drug repositioning and new drug discovery through similarity learning from drug-target joint feature spaces
Source: BMC Bioinformatics. 2019 Dec 27;20(Suppl 23):605. doi: 10.1186/s12859-019-3238-y (PMC6933655; doi:10.1186/s12859-019-3238-y)
Supplement: Supplementary file 2 — Additional file 2 The supplementary figures for this work. ∙ Figure S1: The AUC scores of four classifiers on reliable negative samples with different negative sample ratio levels. ∙ Figure S2: F1-scores of the proposed method with different PCNs (principle component numbers). The x-axis is the PCA component number and the y-axis is the F1-score. [file 12859_2019_3238_MOESM2_ESM.pdf]

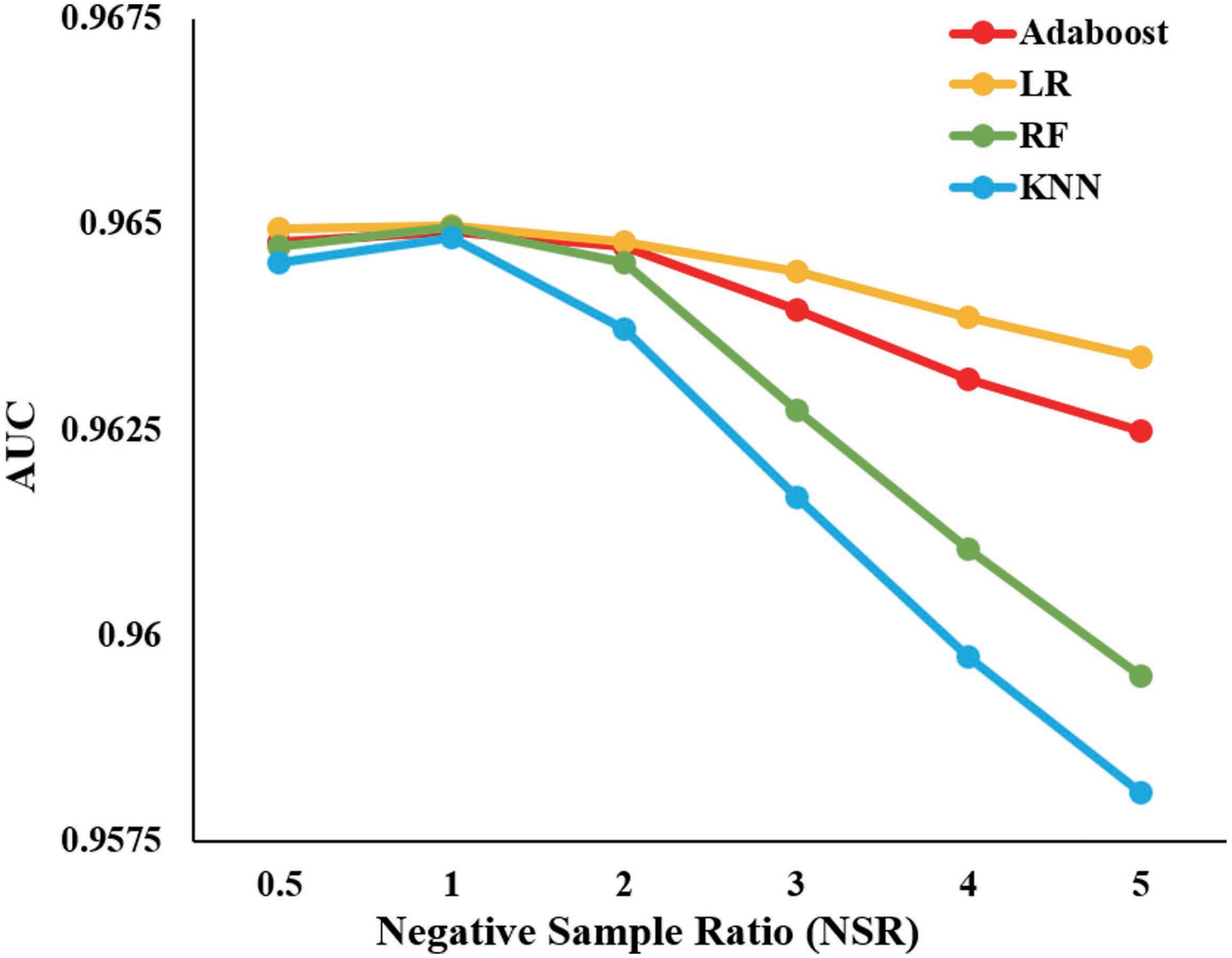

Figure S1. The AUC scores of four classifiers on reliable negative samples with different negative sample ratio levels.

We tuned the PCN (principle component number) with the following settings: PCN∈{1,5,10,15,20,25,30,35,40,45,50,55, 60,65,70,80,85,90,95,110,125,140,150,160,175,200,225,250,275,300,350,400,450,500,550,600,750,800,1000,1250,1750,2000}. It can be observed that the F1-scores for all classifiers increase with PCN at the beginning (1⩽ PCN⩽ 225). When PCN exceeds 225, the F1-Scores plateau for Adaboost and LR while slightly decrease for KNN and RF. Based on the above observation and considering the time-cost (computational time increases with PCN), we set PCN as 225.

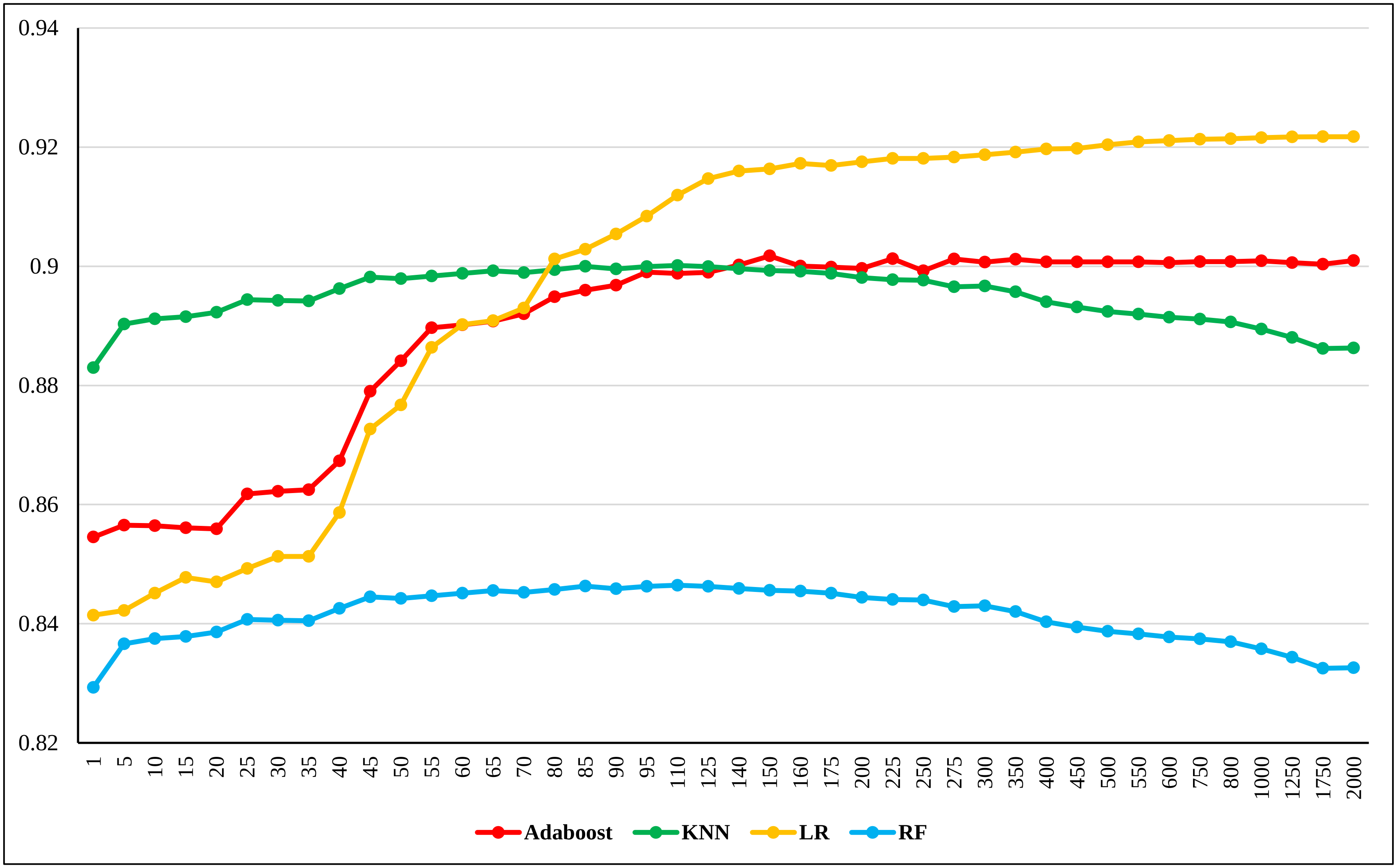

Figure S2. F1-scores of the proposed method with different PCNs (principle component numbers). The x-axis is the PCA component number and the y-axis is the F1-score.
